# FGFR2 protein expression in breast cancer: nuclear localisation and correlation with patient genotype

**DOI:** 10.1186/1756-0500-4-72

**Published:** 2011-03-21

**Authors:** Amy J Martin, Andrew Grant, Alison M Ashfield, Colin N Palmer, Lee Baker, Philip R Quinlan, Colin A Purdie, Alastair M Thompson, Lee B Jordan, Jonathan N Berg

**Affiliations:** 1 Division of Pathology and Neuroscience, University of Dundee, Ninewells Hospital and Medical School, Dundee, DD1 9SY, UK; 2 Department of Clinical Pathology, Ninewells Hospital and Medical School, Dundee, DD1 9SY, UK; 3 Division of Surgery and Molecular Oncology, University of Dundee, Ninewells Hospital and Medical School, Dundee, DD1 9SY, UK; 4 Biomedical Research Institute, University of Dundee, Dundee, DD1 9SY, UK

## Abstract

**Background:**

Single Nucleotide Polymorphisms (SNPs) in intron 2 of the Fibroblast Growth Factor Receptor Type 2 (*FGFR2*) gene, including rs2981582, contribute to multifactorial breast cancer susceptibility. The high risk polymorphism haplotype in the *FGFR2 *gene has been associated with increased mRNA transcription and altered transcription factor binding but the effect on FGFR2 protein expression is unknown. 40 breast tumours were identified from individuals with known rs2981582 genotype. Tumour sections were stained for FGFR2 protein expression, and scored for nuclear and cytoplasmic staining in tumour and surrounding normal tissue.

**Findings:**

FGFR2 immunohistochemistry demonstrated variable nuclear staining in normal tissue and tumour tissue, as well as consistent cytoplasmic staining. We did not find an association between nuclear staining for FGFR2 and genotype, and there was no association between FGFR2 staining and estrogen or progestogen receptor status. There was an association between presence of nuclear staining for FGFR2 in normal tissue and presence of nuclear staining in the adjacent tumour (Fishers exact test, p = 0.002).

**Conclusions:**

Variable nuclear staining for FGFR2 in breast cancer, but an absence of correlation with rs2981582 genotype suggests that the mechanism of action of polymorphisms at the *FGFR2 *locus may be more complex than a direct effect on mRNA expression levels in the final cancer. The effect may relate to FGFR2 function or localisation during breast development or tumourigenesis. Nuclear localisation of FGFR2 suggests an important additional role for this protein in breast development and breast cancer, in addition to its function as a classical cell surface receptor.

## Background

Fibroblast growth factor receptor type 2 (FGFR2) is a receptor tyrosine kinase involved in a number of cell signalling pathways that contribute to cell growth and differentiation [1]. FGFR2 is important in development of a number of tissues including breast and kidney [2,3]. Mutations in *FGFR2 *can also cause rare monogenic diseases such as lacrimo-auriculo-dento-digital (LADD) syndrome and syndromic craniosynostosis [4]. However, recent interest has focussed on Single Nucleotide Polymorphisms (SNPs) in intron 2 of *FGFR2 *, including rs2981582, which form a high risk haplotype that is associated with an increased risk of breast cancer [5,6]. The risk haplotype in *FGFR2 *is associated with both oestrogen receptor positive (ER+ve) and ER-ve tumours, although the association with ER+ve tumours is stronger.

Although principally considered a cell surface receptor, FGFR2 is also found to have nuclear localisation in a number of different breast and cancer related contexts [7,8]. The related receptor FGFR1 has been shown to be transported actively to the nucleus, where it may act as a transcription factor coordinating gene expression and promoting cellular differentiation [9]. FGFR2 has also been has been identified in the terminal end buds during mammary gland development, with nuclear localisation [8], suggesting that the nuclear localisation plays an important role in breast development.

Despite the clear evidence of involvement of FGFR2 in breast cancer, and association of polymorphisms in *FGFR2 *with breast cancer [5,6], the mechanism by which these polymorphisms predispose to breast cancer remains uncertain. Previous analysis of polymorphisms in the *FGFR2 *linkage disequilibrium block including rs2981582 has identified a putative oestrogen receptor binding site and a further polymorphism in a POU transcription factor binding domain [5]. One study has investigated the effect of *FGFR2 *genotype on transcription factor binding and mRNA production [10]. This study demonstrated that the high risk SNP haplotype has higher affinity for the transcription factor RUNX2. However, analysis and quantification of the *FGFR2 *transcript did not show any variation in splicing with genotype, but suggested increased transcription of *FGFR2 *from the minor (increased risk) haplotype.

Further investigation of the *FGFR2 *gene region has suggested that the effect on RUNX2 binding is caused by the polymorphism rs2981578. Other polymorphisms in the same linkage disequilibrium block have been suggested as having an effect on binding of other transcription factors, including rs7895676 which is located in a C/EBPβ binding site [5,11]. One of the effects of polymorphisms in the linkage disequilibrium block appears to be alteration local histone acetylation in breast cancer cell lines [12]. It remains unclear as to whether a single polymorphism is responsible, or the effect is caused by several polymorphisms in *cis *acting together.

Several studies investigating expression of FGFR2 protein in breast cancer seem to contradict the hypothesis that overexpression of FGFR2 is a step in tumour development. Early studies have shown expression of FGFR2 only in a minority (4% and 12%) of breast cancers [13,14]. A more recent study using a combination of different techniques to investigate gene expression, protein levels and genomic changes involving *FGFR2 *in breast cancer suggested that FGFR2 levels are lower in tumour tissue than the adjacent normal breast ducts, and that in a proportion of cases, this could be attributed to LOH or methylation involving the FGFR2 locus [12]. This study did not investigate whether genotype itself had an effect on gene and protein expression.

In order to further investigate the role of FGFR2 expression in breast cancer, and whether protein expression and sub-cellular localisation in tumour correlated with genotype in patients, we used immunohistochemistry to examine FGFR2 expression and localisation in cancers from individuals of known rs2981582 genotype.

## Materials and methods

40 invasive (ductal) carcinomas of no special type were chosen for availability of material for staining, comparable grade, ER, progesterone receptor and HER2 receptor status. Patients were selected as homozygous for the low risk (G) allele (16 patients), heterozygous (A/G) (10 patients), or homozygous for the high risk (A) allele (14 patients). Patient genotype was determined using blood from peripheral blood leucocytes. Ethical approval was obtained through the ethics committee of the Tayside Tissue Bank.

Genotyping of samples had previously carried out using pre-designed Taqman SNP genotyping assays purchased from Applied Biosystems™, carried out according to manufacturer's guidelines and analysed in a 96 well or 384 well format using an ABI 7700™.

Immunohistochemistry was carried out on formalin fixed, paraffin embedded breast cancer tissue. 4 micron sections were placed onto positively charged slides (BDH Lab systems), incubated for 1 hour at 60°C, de-waxed and re-hydrated prior to microwave antigen retrieval in citric acid buffer (citric acid, 10 mM, pH6.4) for 15 minutes. *FGFR2 *expression was detected using a rabbit polyclonal anti-*FGFR2 *antibody (C-17-SC122; Santa Cruz). Negative controls were carried out by omitting the primary antibody. Positive controls included normal skin and a dermatofibroma. Prior to antibody binding, a manual avidin and biotin blocking step was carried out to reduce background. Immunohistochemistry was performed using a TECHMATE™500 autostainer (DAKO). Slides were labelled with the primary antibody (at 1:50 concentration) (1 hr), followed by detection with biotinylated secondary antibody (25 minutes) and streptavidin peroxidase (25 minutes), with peroxidase staining using a solution containing DAB, HRP-substrate buffer and the substrate working solution CHROM (3 × 5 minutes) with wash steps between. Cells were counterstained with haematoxylin and images captured by our Virtual Microscopy (VM) apparatus (Aperio ScanScope XT™, Aperio Technologies), at a x40 objective and archived within the 'Aperio Spectrum Plus + TMA' database (version 9.0.748.1521).

Immunohistochemical staining was reviewed in detail and scored by a single experienced breast pathologist (LBJ) who was blinded to tumour genotype and the scoring was conducted in isolation and in the absence of any other data. A second, blinded, review and opinion by another experienced breast pathologist (CAP) was conducted for corroboration purposes. All staining was checked both using images generated by the Aperio ScanScope™and optically using a Nikon Eclipse E600 microscope.

Initially, invasive malignancy was scored. Subsequently, where present on the original slide, normal breast epithelium was also scored, with additional qualitative comments made on other regions of interest in the slide. Antibody staining was assessed and scored using the "Quick Score method" [15]. Briefly, the proportion of positive cells was estimated and given a score on a scale of 1 to 6; 0 - 4% = 1, 5 - 19% = 2, 20 - 39% = 3, 40 - 59% = 4, 60 - 79, and 80 - 100% = 6. The average intensity of the positively staining cells was estimated and given a score of 0 to 3; no staining = 0, weak staining = 1, intermediate staining = 2, and strong staining = 3. The Quick Score was then calculated by multiplying the percentage of cells staining score by the intensity score to give a maximum value of 18. In addition, the cellular localisation of the antibody staining was noted to be both nuclear and cytoplasmic, and therefore both compartments were scored. No membrane specific staining was present. This score was used for all subsequent analyses.

Data was stored on an Excel™spreadsheet, and analysed by construction of 2 × 2 and 3 × 2 contingency tables, using Fishers exact test, the Chi Square test or Chi square test for trend as appropriate, using OpenEpi 2.2.1.

There is no agreed threshold for scoring FGFR2 staining by immunohistochemistry. For all comparisons with staining, we analysed our data twice, once using a grouping of no staining against any staining (score 0 or greater than 0), and secondly using a cutoff of 0-3 or greater than 3, the same criteria used for estrogen receptor positivity.

Immunohistochemical staining was available on all 40 tumours, but only for 29 normal tissues present in the same slide. For comparisons of tumour with normal tissue, we performed the analysis twice, once using just the 29 tumours with associated normal tissue, and subsequently comparing staining levels in all 40 tumours against all 29 normal tissues.

## Results

**Figure 1:**
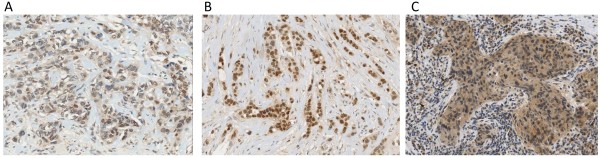
**FGFR2 staining in 3 different intraductal breast cancers of no specific type (all at 20X objective)**. 1A showing occasional nuclear staining. 1B showing prominent staining of all nuclei. 1C showing moderate cytoplasmic staining.

**Figure 2:**
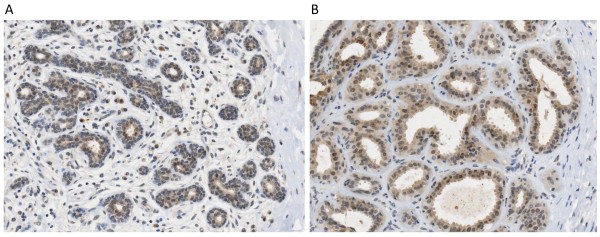
**FGFR2 expression in non-malignant breast epithelium**. Both using 20X objective. 2A showing some nuclear staining in epithelial cells in breast lobule. 2B showing more prominent nuclear staining and some cytoplasmic staining in breast epithelium showing columnar cell features.

Examples of the different patterns of cytoplasmic and nuclear staining for *FGFR2 *that we observed in malignancies and normal tissue are shown in Figures 1 and 2. All except one tumour (which had a G/G genotype) showed faint generalised cytoplasmic staining, which was not confined to the membrane. Therefore, no significant association of genotype with cytoplasmic staining was demonstrated.

**Figure 3:**
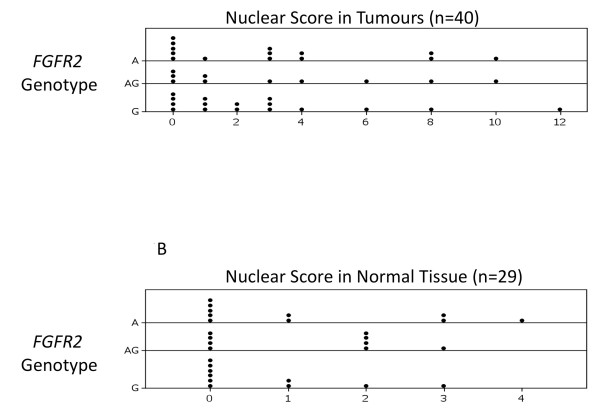
**Dot plots showing distribution of nuclear staining by genotype**. Figure 3A shows results for nuclear staining in tumour and figure 3B shows results for nuclear staining in normal tissue.

More notably, a proportion of tumours showed definite nuclear staining for FGFR2. We therefore compared rs2981582 genotype with nuclear staining for protein. No significant difference was observed in FGFR2 staining between patients with different genotypes at this locus, either using a cut off score of 0, or using a cut off score of 4 (the same score that would be used to assess oestrogen receptor positivity). These data are shown in table 1 and Figure 3.

**Table 1:** Breast Tumour Staining for FGFR2 by Patient Genotype.

rs2981582 genotype	Nuclear Score 0	Nuclear Score >0	Nuclear Score 0-3	Nuclear Score 4+
GG	4	12	12	4

GA	3	7	6	4

AA	5	9	9	5
	χ^2 ^for trend = 0.19, ns		χ^2 ^for trend = 0.69, ns	

A comparison of tumour staining scores by patient genotype. Scores are compared for the 40 tumour samples. No significant difference in staining was found by genotype. The raw data for this analysis is shown in a dot plot in Figure 3.

The normal background breast epithelium (ductal and lobular) could be analysed in 29 cases. Normal epithelium displayed similar cytoplasmic staining, scoring between 3 and 6 in all cases examined. This normal epithelium also displayed variable, often weak and scattered nuclear staining. Stronger nuclear staining was noted in some hyperplastic regions and in areas displaying columnar cell features. No significant difference in FGFR2 staining of normal tissue was observed with rs2981582 genotype, shown in table 2.

**Table 2:** Staining for FGFR2 in Normal Breast Tissue.

rs2981582 genotype	Nuclear Score 0	Nuclear Score >0	Nuclear Score 0-3	Nuclear Score 4+
GG	6	4	10	0

GA	4	5	9	0

AA	5	5	9	1
	χ^2 ^for trend = 0.48, ns		χ^2 ^for trend not calculated	

Staining for FGFR2 in normal tissue where it was present in the same section as tumour. This analysis was possible in 29 samples. No significant difference in staining was found by genotype. The raw data for this analysis is shown in a dot plot in Figure 3.

Analysis of FGFR2 nuclear staining in relation to oestrogen receptor and progesterone receptor status did not show any significant correlation, shown in table 3.

**Table 3:** A comparison of FGFR2 staining with oestrogen and progesterone receptor status of tumours.

	Nuclear Score 0	Nuclear Score >0	Nuclear Score 0-3	Nuclear Score 4+
ER +ve	9	13	17	5

ER -ve	4	14	10	8

	p = 0. 36 (Fishers Exact Test)		p = 0.26 (Fishers Exact Test)	

PR +ve	4	10	10	4

PR -ve	8	17	16	9

	p = 1.0 (Fishers Exact Test)		p = 0.92 (Fishers Exact Test)	

No significant difference in FGFR2 staining was seen with hormonal status of tumours.

We noted that the nuclear staining scores for tumours were sometimes higher than those for the adjacent normal tissue. Analysis of this using a cut-off of less than 4 against a score of 4 or greater suggested that tumours were more likely to show significant nuclear expression of FGFR2 than the surrounding tissue, shown in table 4. This result was marginally significant if we only included tumour from the 29 slides where normal tissue was also present. The significance level was higher if we combined the data to perform a comparison of staining of all 40 tumours and the staining of 29 areas of normal tissue.

**Table 4:** Nuclear FGFR2 Staining in Breast Tumours compared to Normal Tissue

	Nuclear Score 0	Nuclear Score >0	Nuclear Score 0-3	Nuclear Score 4+
Normal Tissue	15	14	28	1

Tumour (normal present)	11	18	22	7

	p = 0.43 (Fishers Exact Test)		p = 0.05 (Fishers Exact Test)	

Tumour (All)	12	28	27	13

	p = 0.12 (Fishers Exact Test)		p = 0.005* (Fishers Exact Test)	

A comparison of FGFR2 staining in normal tissue and tumour tissue. The analysis is performed twice, firstly for a cut-off of 0 (no staining), and secondly for staining scores of 4 or greater. Initially the analysis was performed using data from the 29 cases where both tumour and normal tissue were present in the same slide. Subsequently, we also repeated the analysis using data from all 40 tumours compared to the 29 normal tissues that could be scored. When using a cut-off of 4, the first analysis showed marginal significance using a Fishers exact test, with a higher level of significance seen if all the data was included. This suggests increased FGFR2 staining in a proportion of tumours compared to normal tissue.

A further analysis was performed looking at the normal tissue and tumour tissue for each slide where both were present. This, shown in table 5, showed that in all except one case, where nuclear staining was present at any level in normal tissue, it was present in adjacent tumour. In 5/15 cases, staining was seen in tumour where it was not seen in adjacent normal tissue (p = 0.002, Fishers exact test). This suggests that FGFR2 expression and localisation in tumour tends to reflect the expression and localisation in the background tissue it arises from. However, tumorigenesis may be more likely to increase FGFR2 expression above the background than decrease it.

**Table 5:** A comparison of FGFR2 staining between tumour and adjacent normal tissue within the same slide.

		Nuclear Score of Adjacent Normal Tissue	
		
		0	>0
Tumour Nuclear Score	0	10	1
	
	>0	5	13

		P = 0.002 (Fishers Exact test)	

§		Nuclear Score of Adjacent Normal Tissue	
		
		0-3	4+

Tumour Nuclear Score	0-3	21	1
	
	4+	7	0

		P = 1 (Fishers Exact test)	

A comparison of nuclear staining for FGFR2 within each slide for the 29 samples where normal and tumour tissue was available on the same slide. The first analysis used a cutoff of staining against no staining. In most cases, where staining was present in the normal tissue, this was reflected in the adjacent tumour. In 5 cases, tumour staining was seen where staining of adjacent normal tissue was not, and in only one case did staining appear to be lost in tumour. Repeating this analysis as before, using a cutoff of 4, the analysis was not significant, as only a single normal tissue stained to this level.

Whilst the nuclear staining was quite dramatic, particularly within certain invasive tumours, consideration has been given to the possiblility that the cytoplasmic staining may be a background artefact of this antibody clone, however, it is variable in distribution and intensity and therefore expected to represent actual expression. The nuclear staining for FGFR1 and FGFR2 seen in this and other studies suggests that nuclear staining is a genuine finding for FGFR proteins.

## Discussion

While increased levels of *FGFR2 *transcript have been observed previously in patients who are homozygous for the rare rs2981582 allele [10], we were unable to demonstrate an increase in cytoplasmic protein levels by immunohistochemistry. Although nuclear expression of FGFR2 might represent increased expression of protein, there are other explanations, including altered cellular localisation of the existing protein in the cell. Whichever is the case, no clear correlation with rs2981582 genotype was observed.

The *FGFR2 *mRNA may be unstable, and degraded before translation, so that increased levels do not lead to increased protein production. However, in this case it would be difficult to explain how increased mRNA production would lead to an increased risk of cancer.

Our sample size is comparatively small. For a highly variable cellular phenotype such as protein expression, it is possible that analysis of a larger cohort would reveal a subtle correlation that we were not able to detect.

It is, as yet, uncertain which polymorphism is genuinely responsible for the increased risk, or whether several polymorphisms in *cis *act in concert. Comparison with other polymorphisms in *FGFR2 *might demonstrate a better correlation with protein expression. However, rs2981582 is within the linkage disequilibrium block that confers risk and has originally been shown to correlate with mRNA expression [10].

FGFR2 is involved in breast development [8], so increased *FGFR2 *expression may be an early event in embryonic development or childhood that sets the scene for a higher risk of breast cancer in later life without being involved directly in tumour behaviour in the adult. Breast epithelial cells with an increased risk FGFR2 genotype may be primed to respond differently to growth factors and endocrine influences. Alternatively there may be significant variation in levels of FGFR2 in mature breast, below that detectable by immunohistochemistry. The variation in FGFR2 expression that is responsible for risk may be in a small subpopulation of cells (for example the stem cell precursors), that would not be apparent on conventional viewing of stained sections. It is also possible that the effect of FGFR2 protein expression is mediated by another cell type, such as fibroblasts, affecting the tumour cell microenvironment, not the tumour cells themselves.

In mammary epithelial cells, the specificity of pathway activation by FGFR2 may be dependent on the balance of the different splice forms of this gene [16,17]. The causative polymorphism in *FGFR2 *may affect *FGFR2 *splicing, leading to splice forms that may behave differently in terms of signalling activation or cellular localisation, but which stain similarly by immunohistochemistry.

Although marked overexpression of FGFR2 has previously been reported in up to 12% of breast tumours [13,14], we did not observe this phenomenon in the 40 tumours that we analysed. We did not, therefore, find any evidence that overexpression of FGFR2 was related to genotype. If overexpression of FGFR2 in breast cancer was related to genotype, it is doubtful whether overexpression of FGFR2 in 12% of tumours, even if only possible on a high risk allele background, would in itself, account for the increase in clinical risk in breast cancer. One mechanism for over-expression of FGFR2 would be increase in copy number of *FGFR2 *in tumours. We did not assess this, but the absence of correlation between protein expression and patient constitutional genotype suggests that likelihood of *FGFR2 *gene amplification is not strongly related to patient genotype at the locus.

Nuclear staining for FGFR2 and its importance in human breast cancer has been highlighted by this study. This nuclear expression was variable but more prominent in certain tumours. This has not previously been investigated in human breast cancers, or considered in relation to genotype. The nuclear localisation of FGFR2 raises important questions about the function of the receptor and, hence, the potential mechanism of action of polymorphisms in the gene.

Nuclear expression of FGFR2 has been observed previously in normal mouse breast development and lung tumours [18], as well as mouse mammary gland [8] but not human breast cancer. It seems likely that FGFR2 has a similar, unusual, mechanism of receptor action to FGFR1 [9] which has been shown to localise to the nucleus in some situations, possibly promoting cell differentiation. The nuclear signalling by FGFR2 may, therefore, contribute to a protective mechanism against cancer. It would be important to consider this when designing new breast cancer treatments that target the FGFR2 signalling pathway, as inhibitors of FGFR2 might, therefore, be expected to have the wrong effect on disease progression.

The association between tumour nuclear FGFR2 expression and expression in the original normal tissue is interesting, as it suggests that the level of FGFR2 expression may be programmed into the normal tissue in advance of the cancer developing. As the tissue we examined was on the same slide as the tumour, this may be a regional phenomenon, and a step in tumorigenesis in the breast. Alternatively nuclear FGFR2 expression may be an innate general characteristic of the breast tissue in some individuals, but not others. The factors that control this expression phenotype are therefore interesting in understanding how the control of FGFR2 expression relates to tumour formation and genotype.

There is a disparity between our data, and that of Zhu et al. [12]. We do not see any evidence of down regulation of FGFR2 compared to normal tissue. In addition, we did not see the increase in protein with genotype that might be expected from the data presented by Meyer [10], where increased transcription was seen with the risk genotype, However, as discussed above, there are a number of reasons why increased mRNA expression may not translate into increased protein seen on immunohistochemical staining. Further work is required to establish the effect of *FGFR2 *polymorphisms on gene function.

Our findings, including the nuclear localisation of FGFR2 and the lack of effect of *FGFR2 *genotype at rs2981582 on cytoplasmic expression, are important findings that show that expression of FGFR2 in breast cancer, and its relationship to genotype is complex. More studies are required to understand how the low-penetrance breast cancer risk polymorphisms in *FGFR2 *act to confer this risk, and how this information can be used to improve breast cancer prevention or treatment.

## Competing interests

The authors declare that they have no competing interests.

## Authors' contributions

AM Identified the cases for study, analysed the data and participated in drafting and finalising the manuscript. AG optimised and performed the immunohistochemistry. AA collected the clinical data for cases. AT was involved in provision of tumour and DNA samples, and in project design. C. Purdie assisted in analysis of immunohistochemistry. C. Palmer assisted in sample genotype analysis. LB contributed to the statistical analysis. PQ assisted with data collection and slide imaging. LJ was responsible for immunohistochemical analysis and involved in result interpretation. JB assisted in project design, data interpretation and drafting the manuscript. All authors have read and approved the final manuscript.

## References

[B1] Eswarakumar VP Lax I Schlessinger J Cellular signaling by fibroblast growth factor receptors Cytokine Growth Factor Rev 2005 16 139 49 10.1016/j.cytogfr.2005.01.001 15863030

[B2] Bates CM Role of fibroblast growth factor receptor signaling in kidney development Pediatr Nephrol 2007 22 343 9 10.1007/s00467-006-0239-7 16932896

[B3] Parsa S Ramasamy SK De Langhe S Gupte VV Haigh JJ Medina D Bellusci S Terminal end bud maintenance in mammary gland is dependent upon FGFR2b signaling Dev Biol 2008 317 121 31 10.1016/j.ydbio.2008.02.014 18381212

[B4] Wilkie AO Bochukova EG Hansen RM Taylor IB Rannan-Eliya SV JC Wall SA Ramos L Venâncio M Hurst JA O'rourke AW Williams LJ Seller A Lester T Clinical dividends from the molecular genetic diagnosis of craniosynostosis Am J Med Genet A 2007 143A 1941 9 10.1002/ajmg.a.31905 17621648

[B5] Easton DF Pooley KA Dunning AM Pharoah PD Thompson D Ballinger DG Struewing JP Morrison J Field H Luben R Wareham N Ahmed S Healey CS Bowman R SEARCH collaborators Meyer KB Haiman CA Kolonel LK Henderson BE Le Marchand L Brennan P Sangrajrang S Gaborieau V Odefrey F Shen CY Wu PE Wang HC Eccles D Evans DG Peto J Fletcher O Johnson N Seal S Stratton MR Rahman N Chenevix-Trench G Bojesen SE Nordestgaard BG Axelsson CK Garcia-Closas M Brinton L Chanock S Lissowska J Peplonska B Nevanlinna H Fagerholm R Eerola H Kang D Yoo KY Noh DY Ahn SH Hunter DJ Hankinson SE Cox DG Hall P Wedren S Liu J Low YL Bogdanova N Schürmann P Dörk T Tollenaar RA Jacobi CE Devilee P Klijn JG Sigurdson AJ Doody MM Alexander BH Zhang J Cox A Brock IW MacPherson G Reed MW Couch FJ Goode EL Olson JE Meijers-Heijboer H van den Ouweland A Uitterlinden A Rivadeneira F Milne RL Ribas G Gonzalez-Neira A Benitez J Hopper JL McCredie M Southey M Giles GG Schroen C Justenhoven C Brauch H Hamann U Ko YD Spurdle AB Beesley J Chen X AOCS Management Group Mannermaa A Kosma VM Kataja V Hartikainen J Day NE Cox DR Ponder BA Genome-wide association study identifies novel breast cancer susceptibility loci Nature 2007 447 1087 93 10.1038/nature05887 17529967 PMC2714974

[B6] Hunter DJ Kraft P Jacobs KB Cox DG Yeager M Hankinson SE Wacholder S Wang Z Welch R Hutchinson A Wang J Yu K Chatterjee N Orr N Willett WC Colditz GA Ziegler RG Berg CD Buys SS McCarty CA Feigelson HS Calle EE Thun MJ Hayes RB Tucker M Gerhard DS Fraumeni JF Jr Hoover RN Thomas G Chanock SJ A genome-wide association study identifies alleles in FGFR2 associated with risk of sporadic postmenopausal breast cancer Nat Genet 2007 39 870 4 10.1038/ng2075 17529973 PMC3493132

[B7] Giulianelli S Cerliani JP Lamb CA Fabris VT Bottino MC Gorostiaga MA Novaro V Góngora A Baldi A Molinolo A Lanari C Carcinoma-associated fibroblasts activate progesterone receptors and induce hormone independent mammary tumor growth: A role for the FGF-2/FGFR-2 axis Int J Cancer 2008 123 2518 31 10.1002/ijc.23802 18767044

[B8] Lu P Ewald AJ Martin GR Werb Z Genetic mosaic analysis reveals FGF receptor 2 function in terminal end buds during mammary gland branching morphogenesis Dev Biol 2008 321 77 87 10.1016/j.ydbio.2008.06.005 18585375 PMC2582391

[B9] Stachowiak MK Maher PA Stachowiak EK Integrative nuclear signaling in cell development--a role for FGF receptor-1 DNA Cell Biol 2007 26 811 26 10.1089/dna.2007.0664 18021009

[B10] Meyer KB Maia AT O'Reilly M Teschendorff AE Chin SF Caldas C Ponder BA Allele-Specific Up-Regulation of FGFR2 Increases Susceptibility to Breast Cancer PLoS Biol 2008 6 e108 10.1371/journal.pbio.0060108 18462018 PMC2365982

[B11] Zhu X Asa SL Ezzat S Histone-acetylated control of fibroblast growth factor receptor 2 intron 2 polymorphisms and isoform splicing in breast cancer Mol Endocrinol 2009 23 1397 405 10.1210/me.2009-0071 19497954 PMC2737561

[B12] Zhu X Asa SL Ezzat S Genetic and epigenetic mechanisms down-regulate FGF receptor 2 to induce melanoma-associated antigen A in breast cancer Am J Pathol 2010 176 2333 43 10.2353/ajpath.2010.091049 20348248 PMC2861098

[B13] Adnane J Gaudray P Dionne CA Crumley G Jaye M Schlessinger J Jeanteur P Birnbaum D Theillet C BEK and FLG, two receptors to members of the FGF family, are amplified in subsets of human breast cancers Oncogene 1991 6 659 63 1851551

[B14] Penault-Llorca F Bertucci F Adelaide J Parc P Coulier F Jacquemier J Birnbaum D deLapeyrière O Expression of FGF and FGF receptor genes in human breast cancer Int J Cancer 1995 61 170 6 10.1002/ijc.2910610205 7705943

[B15] Detre S Saclani Jotti G Dowsett M A "quickscore" method for immunohistochemical semiquantitation: validation for oestrogen receptor in breast carcinomas J Clin Pathol 1995 48 9 876 8 10.1136/jcp.48.9.876 7490328 PMC502883

[B16] Kondo T Zheng L Liu W Asa SL Ezzat S Epigenetically controlled fibroblast growth factor receptor 2 signaling imposes on the RAS/BRAF/mitogen-activated kinase pathway to modulate thyroid cancer progression Cancer Res 2007 67 5461 70 10.1158/0008-5472.CAN-06-4477 17545628

[B17] Moffa AB Tannheimer SL Ethier SP Transforming potential of alternatively spliced variants of fibroblast growth factor receptor 2 in human mammary epithelial cells Mol Cancer Res 2004 2 11 643 52 15561780

[B18] Behrens C Lin HY Lee JJ Raso MG Hong WK Wistuba II Lotan R Immunohistochemical expression of basic fibroblast growth factor and fibroblast growth factor reeptors 1 and 2 in the pathogenesis of lung cancer Clin Cancer Res 2008 14 19 6014 22 10.1158/1078-0432.CCR-08-0167 18829480 PMC5108626

